# Sex-Related Differences in Suspected Pediatric Anaphylaxis in a Tertiary Hospital Between 2015 and 2022: A Retrospective Cross-Sectional Study and Implications for Nursing Practice

**DOI:** 10.3390/nursrep16090310

**Published:** 2026-09-01

**Authors:** Ana Fernandez-Alonso, Victoria Mateo-Frances, Lucia Noguerales-Fuertes, Alejandra Jover-Walsh, Juan Sebastian Arana-Arce, Paula Cubillo-Heras, Victor Fernandez-Alonso

**Affiliations:** 1 Fundación para la Investigación Biomédica del Hospital Gregorio Marañón, 28007 Madrid, Spain; 2 Department of Allergy, Gregorio Marañón General University Hospital, 28007 Madrid, Spain; 3 Nursing Care Research Group, Gregorio Marañón Health Research Institute (IiSGM), 28007 Madrid, Spain; 4 Red Cross University School of Nursing, Universidad Autónoma de Madrid, 28003 Madrid, Spain

**Keywords:** anaphylaxis, pediatrics, sex, allergy and immunology, suspected triggers, nursing

## Abstract

**Background/Objectives**: Anaphylaxis is a severe systemic allergic reaction that may involve multiple organ systems and requires prompt recognition and treatment. In children and adolescents, food is a major trigger, and clinical presentation may vary according to biological factors, including sex. This study aimed to examine sex-related differences in the clinical presentation, triage acuity, measured emergency-care processes, and suspected triggers of anaphylaxis in pediatric patients treated at a tertiary hospital. **Methods:** A retrospective cross-sectional study was conducted in the pediatric emergency department of a tertiary hospital in Madrid, Spain, between 2015 and 2022. Sociodemographic characteristics, allergic history, clinical manifestations, TRIPED-GM triage level, Pediatric Assessment Triangle classification, measured emergency-care processes, suspected triggers, hospital admission, and final diagnosis were analyzed. Multiple comparisons were controlled using Bonferroni adjustment within clinically defined domains. **Results:** A total of 368 patients were included; 65.2% were boys and 57.3% were aged ≤5 years. Unadjusted analyses identified several apparent signals in specific clinical manifestations, triage distribution, and suspected triggers; none showed a consistent pattern after multiplicity adjustment. The difference in the overall distribution of TRIPED-GM triage levels was not statistically significant after multiplicity adjustment. Although Grade 3 assignment remained more frequent among boys in a category-specific post hoc analysis, this isolated finding was not supported by multivariable ordinal regression, in which sex was not independently associated with overall triage acuity. Tree nuts, cow’s milk protein, and egg were the most frequently suspected triggers. **Conclusions:** No consistent evidence of overall sex-related differences in suspected pediatric anaphylaxis presentation, measured emergency-care processes, suspected triggers, or triage acuity was found after multiplicity adjustment and multivariable analysis. Although an isolated difference in Grade 3 triage assignment persisted in a post hoc analysis, it should be interpreted cautiously. Pediatric emergency nursing assessment and triage should therefore remain primarily guided by acute clinical presentation and physiological severity rather than by sex. Larger multicenter studies are needed to determine whether more subtle or age-dependent sex-related patterns have clinical relevance.

## 1. Introduction

Anaphylaxis is a severe, rapidly evolving systemic hypersensitivity reaction that may be life-threatening and can involve multiple organ systems [1,2]. In pediatric patients, diagnosis is based on the acute onset of compatible clinical manifestations, including skin or mucosal involvement associated with respiratory, cardiovascular, or gastrointestinal compromise, or acute hypotension, bronchospasm, or laryngeal involvement after exposure to a known or potential allergen [1,3]. Prompt recognition is essential because delayed diagnosis and treatment may increase the risk of severe outcomes [1].

The diagnosis of anaphylaxis can be particularly challenging in infants and young children because clinical manifestations may be subtle or nonspecific and may overlap with common findings in this age group [1,4]. Symptoms such as irritability, vomiting, flushing, or behavioral changes may therefore require careful interpretation within the overall clinical context. Consequently, pediatric emergency assessment should integrate the temporal relationship with a potential trigger, the pattern of organ-system involvement, and the child’s physiological status [3,4].

Cutaneous and mucosal manifestations are among the most frequently reported features of pediatric anaphylaxis, commonly accompanied by respiratory and gastrointestinal involvement, whereas cardiovascular manifestations tend to be less prominent in children than in adults [1,4,5]. Food represents a major trigger of pediatric anaphylaxis, with cow’s milk, egg, tree nuts, and other foods accounting for a substantial proportion of reactions during childhood [6,7]. Previous studies conducted in emergency settings have similarly identified food as an important trigger of pediatric anaphylaxis [2,7].

Intramuscular adrenaline is the first-line treatment for anaphylaxis and should be administered promptly when anaphylaxis is clinically recognized [1,3]. In the emergency setting, however, initial nursing assessment and triage precede or occur simultaneously with treatment and depend on rapid identification of airway, breathing, circulatory, cutaneous, and gastrointestinal manifestations and signs of physiological compromise. Structured pediatric assessment and triage are therefore particularly relevant for the early recognition of children who may require urgent intervention.

Biological sex has been proposed as a potential modifier of allergic disease through immunological, hormonal, and mast-cell-related mechanisms [8,9,10]. A greater proportion of boys has been reported in several pediatric allergy and anaphylaxis cohorts, although sex distributions may vary according to age and developmental stage [10,11,12]. Nevertheless, evidence regarding whether sex is independently associated with the clinical presentation or severity of pediatric anaphylaxis remains limited [9,12].

Moreover, reported sex-related associations may be difficult to interpret when numerous clinical outcomes are examined simultaneously without appropriate consideration of multiple testing. Few studies have specifically examined sex-related differences in pediatric patients presenting to emergency departments with suspected anaphylaxis while simultaneously evaluating clinical manifestations, triage acuity, measured emergency-care processes, and suspected triggers. Accordingly, this study aimed to examine sex-related differences in clinical presentation, triage acuity, measured emergency-care processes, and suspected triggers among pediatric patients presenting to a tertiary emergency department with suspected anaphylaxis.

## 2. Materials and Methods

### 2.1. Study Design

This was a retrospective cross-sectional study based on the review of electronic medical records of pediatric patients assessed for suspected anaphylaxis in the pediatric emergency department of a tertiary hospital between 2015 and 2022. The study is reported in accordance with the Strengthening the Reporting of Observational Studies in Epidemiology (STROBE) statement for cross-sectional studies [13].

### 2.2. Criteria

Inclusion criteria were pediatric patients who presented to the pediatric emergency department between 2015 and 2022 with a clinical suspicion of anaphylaxis, based on the presenting symptoms and/or the diagnosis recorded in the medical record. Patients were retained in the cohort regardless of whether they subsequently fulfilled the specific Galaxia diagnostic criteria for anaphylaxis [3].

Exclusion criteria were incomplete clinical records that did not allow assessment of the main study variables, duplicated episodes, or cases in which the initial suspicion of anaphylaxis was subsequently attributed to an alternative diagnosis and anaphylaxis was clinically excluded.

### 2.3. Variables

Study variables were grouped into three categories. First, sociodemographic variables included age and sex. Second, clinical variables included personal and family history of allergic disease, including rhinoconjunctivitis, asthma, food allergy, atopic dermatitis, and previous anaphylaxis. Third, healthcare-process variables included triage level, Pediatric Assessment Triangle (PAT) classification, need for care in the shock/resuscitation room, supplemental oxygen therapy, hospital admission, and suspected trigger.

A separate variable recorded whether each patient fulfilled the Galaxia diagnostic criteria for anaphylaxis after clinical assessment [3]. This variable was used to distinguish patients meeting the specific diagnostic criteria from those who remained clinically suspected cases but did not fulfill all Galaxia criteria. Fulfilment of the Galaxia criteria was assessed by an allergy specialist.

Initial assessment was performed using the Pediatric Assessment Triangle (PAT), a structured pediatric assessment tool that evaluates appearance, work of breathing, and circulation to the skin. The recorded PAT classification included stable, respiratory distress, respiratory failure, compensated shock, decompensated shock, cardiorespiratory compromise, and central nervous system dysfunction [14].

The PAT classification used in this study corresponded to the assessment documented by the clinical team during the initial pediatric emergency evaluation and was not retrospectively reconstructed from individual physiological variables. Although cardiovascular parameters such as blood pressure, heart rate, capillary refill time, and peripheral perfusion may contribute to the clinical assessment of circulatory status, these variables were not consistently available in the retrospective medical records and were therefore not analyzed as independent study variables. Consequently, the PAT categories of compensated and decompensated shock reflected the structured clinical classification recorded at the time of initial assessment rather than a retrospective classification based on individual hemodynamic measurements.

Triage priority was classified using the five-level TRIPED-GM pediatric triage system of Hospital General Universitario Gregorio Marañón [15]. TRIPED-GM information was analyzed according to the triage level documented during the initial emergency department assessment.

Clinical manifestations were classified as cutaneous (urticaria, cutaneous angioedema, and generalized erythema), upper airway/oropharyngeal (pharyngeal angioedema and sialorrhea), respiratory (dyspnea, pharyngeal involvement, pharyngeal obstruction, tachypnea, chest tightness, wheezing, stridor, cyanosis, cough, chest retractions, and dysphonia), gastrointestinal (vomiting, abdominal pain, nausea, and diarrhea), circulatory (dizziness, loss of consciousness, livedo, and diaphoresis), and neurological (irritability, confusion, and hypotonia). Pharyngeal angioedema, pharyngeal involvement, and pharyngeal obstruction were recorded as separate clinical variables. Pharyngeal involvement referred to documented pharyngeal manifestations other than angioedema or obstruction. Patients could present with more than one pharyngeal manifestation when distinct findings were documented in the clinical record.

Gastrointestinal manifestations included vomiting, abdominal pain, nausea, and diarrhea, as documented in the emergency medical record. Because of the retrospective nature of the study, information on the persistence or recurrence of vomiting and the intensity of abdominal pain was not consistently available, and these characteristics could not be analyzed separately. Gastrointestinal symptoms were therefore recorded as clinical manifestations rather than used in isolation to retrospectively establish the diagnosis of anaphylaxis. Fulfilment of the Galaxia diagnostic criteria was recorded separately on the basis of the overall clinical presentation documented during the emergency assessment [3].

Oxygen therapy was recorded as a binary healthcare-process variable according to whether supplemental oxygen administration was documented during the pediatric emergency department episode. Because the retrospective records did not consistently provide the indication for oxygen therapy, oxygen saturation thresholds, delivery device, flow rate, or duration of treatment, these characteristics were not analyzed. Oxygen therapy was therefore considered a measured emergency-care process rather than a marker of anaphylaxis severity or treatment adequacy.

The measured emergency-care processes evaluated in this study were limited to PAT classification, TRIPED-GM triage level, shock/resuscitation-room care, supplemental oxygen therapy, and hospital admission. The study did not comprehensively assess the pharmacological treatment or overall management of anaphylaxis.

Information on previous allergic conditions, including asthma, food allergy, atopic dermatitis, rhinoconjunctivitis, and previous anaphylaxis, was obtained from the medical history documented in the electronic clinical record. These variables reflected previously recorded clinical diagnoses and/or allergic history reported during the emergency department assessment. No retrospective allergological testing was performed for the purposes of this study.

The suspected trigger was identified from the temporal relationship between exposure and symptom onset as reported by the patient or caregiver and documented by the emergency clinician. Because systematic allergological confirmation by skin testing, specific IgE testing, component-resolved diagnostics, or oral food challenge was not available for all episodes, triggers were considered suspected rather than confirmed. Accordingly, the term “suspected trigger” is used throughout the manuscript.

The amount of missing data were assessed separately for each study variable. The number and percentage of available and missing observations are reported in the corresponding tables and in Supplementary Table S1. Analyses were performed using available cases for each comparison, and no statistical imputation of missing values was undertaken. Consequently, denominators may vary across variables and are stated explicitly whenever they differ from the total cohort.

### 2.4. Data Analysis

As this was a retrospective analysis including all eligible cases identified during the study period, no a priori sample-size calculation was performed; the final sample size was therefore determined by the number of eligible cases available in the clinical records.

Data were analyzed using Stata Statistical Software (Release 17, StataCorp LLC, College Station, TX, USA). Continuous variables were assessed for normality and are presented as means with standard deviations or medians with interquartile ranges, as appropriate. Categorical variables are expressed as absolute and relative frequencies. Bivariate comparisons were performed using Student’s *t*-test or the Mann–Whitney U test for continuous variables and Pearson’s chi-square test or Fisher’s exact test for categorical variables, as appropriate.

To control the family-wise Type I error rate across multiple exploratory comparisons, *p*-value adjustments were performed using the Bonferroni method within three clinically distinct domains: (1) allergic history and clinical manifestations, (2) emergency department severity assessment and measured emergency-care processes, and (3) suspected triggers.

Within the emergency department severity and healthcare-process domain, six primary comparisons were considered: overall PAT classification, need for shock/resuscitation-room care, oxygen therapy, final diagnosis of anaphylaxis, hospital admission, and the overall distribution of TRIPED-GM triage levels. Bonferroni adjustment was applied across these six comparisons. Exploratory post hoc comparisons of the five individual TRIPED-GM grades were treated as a separate comparison family and adjusted across the five categories. Both raw and Bonferroni-adjusted *p*-values are reported.

Sex-related differences across the five-level TRIPED-GM triage classification were evaluated using a global Pearson chi-square test or an exact test with Monte Carlo estimation when expected cell counts were <5; Cramér’s V was reported as a measure of effect size.

The independent association between sex and overall triage acuity was further evaluated using ordinal logistic regression, including age as a continuous variable and personal history of food allergy, asthma, and atopic dermatitis as covariates. Adjusted odds ratios (aORs) and 95% confidence intervals (CIs) were estimated. Male sex and the absence of each personal allergic condition were used as reference categories. Statistical significance was defined as a two-tailed *p*-value < 0.05 or below the corresponding Bonferroni-adjusted threshold, as applicable.

### 2.5. Ethical Considerations

The study was approved by the Ethics Committee of Hospital General Universitario Gregorio Marañón (code ALE-ANFX-SUP-1, act 16/2024) and was conducted in accordance with the principles of the Declaration of Helsinki and Regulation (EU) 2016/679 of the European Parliament and of the Council on data protection, ensuring appropriate data coding and anonymization [16].

## 3. Results

A total of 368 patients were included, of whom 240 (65.2%) were boys and 128 (34.8%) were girls. By age: 26.9% (*n* = 99) were <1 year; 30.4% (*n* = 112) were 1–5 years; 20.4% (*n* = 75) were 6–10 years; 21.5% (*n* = 79) were 11–15 years; and 0.8% (*n* = 3) were 16–17 years. The median age was 4 [1,2,3,4,5,6,7,8,9,10] years and 56 [20.5–121.5] months. Significant differences in age were observed: months (*p* = 0.036). During the 2015–2022 study period, the annual rate of included suspected-anaphylaxis episodes per 1000 pediatric emergency department visits varied over time. The annual rate decreased from 1.06 in 2015 to 0.57 in 2016, remained relatively stable between 2017 and 2020 (0.56–0.82), and subsequently increased to 1.25 in 2021 and 1.26 in 2022.

Age and sex data were available for all 368 patients. Completeness varied for some healthcare-process variables. TRIPED-GM triage information was available for 278 patients (75.5%), with 90 observations missing (24.5%), while suspected-trigger information was available for 241 patients (65.5%), with 127 observations missing (34.5%). The completeness of the remaining variables is presented in Table 1, Table 2 and Table 3 and Supplementary Table S1.

Table 1 summarizes the family and personal clinical history of the study population, as well as the main symptoms observed during the anaphylaxis episode. The variables included provide an overview of relevant background factors, such as previous allergic diseases, family history of atopy, and associated comorbidities, together with the clinical manifestations recorded during the acute event. This information allows characterization of the patients’ baseline allergic profile and the pattern of symptom involvement during anaphylaxis.

During the study period, an increase in the number of patients who presented to the pediatric emergency department was observed in recent years. Emergency department assessment, triage acuity, and measured emergency-care processes according to sex are summarized in Table 2.

Although the overall analysis of the TRIPED-GM triage distribution suggested possible differences between boys and girls, these did not remain statistically significant after adjustment for multiple comparisons (χ^2^ = 13.2; *p* = 0.010; *p*-adj = 0.060; Cramér’s V = 0.22). However, when each triage level was analyzed separately, Grade 3 was significantly more frequent in boys than in girls (42.5% vs. 22.7%; OR = 0.40, 95% CI 0.23–0.69), and this difference remained statistically significant after Bonferroni correction (*p*-adj = 0.005).

**Figure 1:**
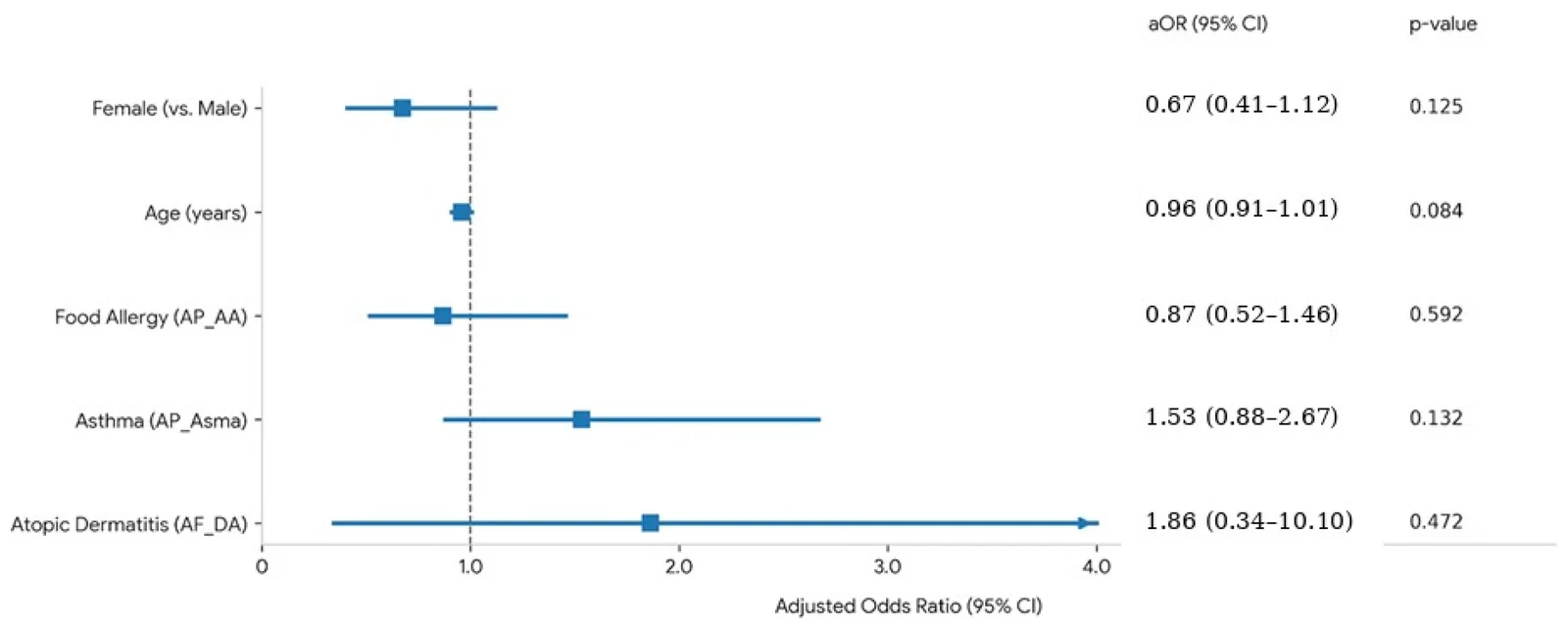
Forest plot of adjusted odds ratios for factors associated with triage severity. Markers represent adjusted odds ratios (aOR) derived from the ordinal logistic regression model (*n* = 278); horizontal error bars depict 95% confidence intervals (95% CI). The vertical dashed line at 1.0 indicates the line of no effect. Reference categories and baseline comparisons are male sex, age in years (cont inuous), and absence of personal clinical history.

A total of 278 patients were included in the ordinal logistic regression analysis. The overall model was not statistically significant (LR χ^2^(5) = 8.91; *p* = 0.113; pseudo-R^2^ = 0.0159). Model estimates and 95% CIs are summarized in Figure 1.

The point estimate suggested lower odds of being assigned to a higher triage severity level among female patients compared with male patients, although the association was not statistically significant (aOR = 0.675, 95% CI: 0.408–1.116, *p* = 0.125). Each additional year of age was associated with a slight non-significant reduction in triage acuity (aOR = 0.957, 95% CI: 0.910–1.006, *p* = 0.084). Regarding clinical background, personal history of food allergy showed no significant association with triage severity (aOR = 0.868, 95% CI: 0.518–1.455, *p* = 0.592). Similarly, point estimates toward higher triage acuity were observed for patients with a personal history of asthma (aOR = 1.532, 95% CI: 0.879–2.668, *p* = 0.132) and a personal history of atopic dermatitis (aOR = 1.861, 95% CI: 0.343–10.097, *p* = 0.472).

### 3.1. Clinical Manifestations During the Episode

Clinical manifestations were analyzed according to the organ systems involved. Cutaneous manifestations were documented in 91.8% of the cohort (338/368). Among patients with cutaneous involvement, urticaria was documented in 61.8% (*n* = 209), cutaneous angioedema in 54.1% (*n* = 183), and generalized erythema in 33.7% (*n* = 114). Because these manifestations were not mutually exclusive, patients could present with more than one cutaneous manifestation. A total of 506 cutaneous manifestations were recorded. Upper airway/oropharyngeal manifestations were infrequent, with pharyngeal angioedema documented in 28 patients and sialorrhea in 4. Respiratory compromise was documented in 75.8% of children (*n* = 279/368), accounting for 490 recorded events. Within this subgroup, 49.5% experienced one symptom, 34.1% two, 11.1% three, 3.6% four, and the remaining 1.8% exhibited between five and seven symptoms. Relative to the total cohort (*n* = 368), dyspnea was the most frequent respiratory manifestation (54.3%, n = 200), followed by wheezing (23.4%, *n* = 86) and cough (19.3%, *n* = 71). Although the unadjusted analysis suggested a higher frequency of pharyngeal involvement among girls (20.3% vs. 12.5%; *p* = 0.040), this association did not remain statistically significant after Bonferroni correction (*p*-adj = 1.000).

Gastrointestinal manifestations were documented in 59.0% of patients (217/368), accounting for 251 recorded manifestations. Among patients with gastrointestinal involvement, 85.7% had one manifestation, 13.4% had two, and 0.5% had three. In the total cohort, vomiting was the most frequent gastrointestinal manifestation (42.7%, *n* = 157), followed by abdominal pain (16.6%, *n* = 61), nausea (5.4%, *n* = 20), and diarrhea (3.5%, *n* = 13). The nominal unadjusted difference observed for diarrhea (*p* = 0.039) did not remain statistically significant after multiplicity adjustment (*p*-adj = 1.000).

Finally, neurological features were identified in 5.4% of the cohort (*n* = 20/368; 23 total reactions), predominantly presenting as irritability (4.1%, *n* = 15). Circulatory symptoms were the least common, documented in 3.8% of patients (*n* = 14/368; 15 total reactions), led by dizziness (1.9%, *n* = 7) and loss of consciousness (1.4%, *n* = 5). No sex-related differences were observed for either system.

Overall, after applying *p*-value adjustments across clinical domains, there was no robust evidence supporting sex-specific differences in symptom presentation.

### 3.2. Emergency Department Assessment, Triage Acuity, and Measured Emergency-Care Processes

Triage priority was evaluated using the TRIPED-GM system in 278 patients with available triage data (181 boys and 97 girls; 24.5% missing). Overall, 1.8% (*n* = 5) were assigned to Grade 1, 53.6% (*n* = 149) to Grade 2, 35.6% (*n* = 99) to Grade 3, 8.6% (*n* = 24) to Grade 4, and 0.4% (*n* = 1) to Grade 5.

The global comparison of the overall distribution of TRIPED-GM levels suggested a difference between boys and girls in the unadjusted analysis (χ^2^ = 13.2, *p* = 0.010), but this association did not remain statistically significant after Bonferroni adjustment across the six primary ED severity and healthcare-process comparisons (*p*-adj = 0.060; Cramér’s V = 0.22). In exploratory category-specific post hoc analyses, adjusted separately across the five TRIPED-GM grades, Grade 3 assignment was more frequent in boys than in girls (42.5% vs. 22.7%; OR for girls vs. boys = 0.40, 95% CI 0.23–0.69), and this difference remained statistically significant after Bonferroni adjustment (*p*-adj = 0.005). No other individual TRIPED-GM category remained statistically significant after adjustment.

In the multivariable ordinal logistic regression, sex was not independently associated with overall triage acuity. Girls had an adjusted odds ratio of 0.675 compared with boys (95% CI 0.408–1.116; *p* = 0.125). The overall regression model was also not statistically significant (LR χ^2^(5) = 8.91, *p* = 0.113; pseudo-R^2^ = 0.0159). Thus, although an isolated Grade 3 difference was identified in the post hoc category-specific analysis, neither the adjusted global comparison nor the ordinal regression provided evidence of a consistent sex-related difference across the overall TRIPED-GM triage distribution.

### 3.3. Analysis of Suspected Triggers

Suspected-trigger information was available for 241 patients (65.5%), including 155 boys and 86 girls. Because suspected-trigger categories were not mutually exclusive, more than one suspected trigger could be recorded for the same patient. Egg was more frequently recorded among boys, whereas shellfish was more frequently recorded among girls in the unadjusted analyses; neither association remained statistically significant after multiplicity adjustment (Table 3).

## 4. Discussion

The present study did not identify a consistent overall pattern of sex-related differences in clinical presentation, triage acuity, measured emergency-care processes, or suspected triggers among children presenting with suspected anaphylaxis after adjustment for multiple comparisons. Although several apparent associations were observed in the unadjusted analyses, most did not remain statistically significant after multiplicity correction. The main exception was the category-specific finding for Grade 3 TRIPED-GM assignment, which remained more frequent among boys after Bonferroni adjustment. However, this isolated finding was not supported by either the adjusted global TRIPED-GM comparison or the multivariable ordinal regression. Taken together, these results do not support sex as a consistent independent determinant of overall emergency triage acuity in this cohort.

Among the 278 patients with available TRIPED-GM data, sex was not independently associated with overall triage acuity after adjustment for age and previous food allergy, asthma, and atopic dermatitis. Although the point estimates suggested lower odds of higher-acuity triage among girls and higher odds among patients with asthma or atopic dermatitis, none of these associations reached statistical significance. Furthermore, the low pseudo-R^2^ indicates that the included background characteristics contributed little to the overall model fit. This finding is consistent with the principles of structured pediatric emergency triage, in which priority is primarily determined by the child’s acute clinical presentation and physiological status rather than by demographic characteristics or non-acute allergic history [15].

The global TRIPED-GM analysis likewise did not provide robust evidence of an overall sex-related difference in triage acuity. Although the unadjusted distribution suggested a difference between boys and girls, the association was no longer statistically significant after adjustment for multiple comparisons. The category-specific post hoc analysis identified a higher frequency of Grade 3 assignment among boys, and this finding remained statistically significant after correction across the five TRIPED-GM categories. Nevertheless, because it was not supported by the global comparison or the multivariable model, it should be interpreted cautiously as an exploratory category-specific finding rather than as evidence of a systematic sex-related difference in pediatric emergency triage.

Boys accounted for 65.2% of the included cohort, with a substantial proportion of participants concentrated in the younger age groups. This distribution describes the composition of the study sample and should not, by itself, be interpreted as evidence of a higher incidence or risk of suspected pediatric anaphylaxis among boys. The high representation of younger children is consistent with the importance of age in the clinical presentation of pediatric anaphylaxis [4]. Moreover, because sex-specific population or pediatric emergency department denominators were not available, the present study cannot determine whether the incidence of suspected pediatric anaphylaxis differs between boys and girls.

Cutaneous manifestations were the most frequent presenting features in our cohort, followed by respiratory and gastrointestinal involvement. This pattern is broadly consistent with previous studies showing that the clinical presentation of pediatric anaphylaxis frequently involves cutaneous, respiratory, and gastrointestinal manifestations, although the relative frequency of individual organ-system involvement varies according to age and study population [4,5]. Gudichsen et al. also reported substantial heterogeneity in the clinical presentation of anaphylaxis in the emergency setting [7]. These observations reinforce the importance of assessing the complete clinical presentation rather than relying on a single symptom or organ system.

In the unadjusted analyses, pharyngeal involvement and diarrhea showed apparent sex-related differences. However, neither association remained statistically significant after Bonferroni correction. These findings should therefore be interpreted as exploratory signals rather than as established sex-specific clinical patterns. Overall, the multiplicity-adjusted analyses did not identify a consistent association between sex and the clinical manifestations recorded in children with suspected anaphylaxis.

Food was the predominant suspected trigger in our cohort, with tree nuts, cow’s milk protein, and egg among the most frequently recorded suspected triggers. This finding is consistent with previous pediatric anaphylaxis studies identifying food as a major elicitor, particularly cow’s milk, egg, and tree nuts [6,7]. Because systematic allergological confirmation was not available for every episode, these exposures should be interpreted as suspected rather than confirmed triggers.

Regarding individual suspected triggers, egg was more frequently recorded among boys in the unadjusted analysis, whereas shellfish was more frequently recorded among girls (OR = 5.74, 95% CI 1.13–29.08). However, neither association remained statistically significant after Bonferroni adjustment (*p*-adj = 0.241 and 0.181, respectively). Previous studies have reported age- and sex-related variation in food allergy and allergen sensitization patterns [17,18,19]. Given the small number of shellfish-related episodes and the wide confidence interval, these findings should be interpreted as exploratory, hypothesis-generating signals that require confirmation in larger prospective pediatric cohorts.

The principal contribution of this study therefore lies not in demonstrating clinically meaningful sex differences, but in showing that several apparently plausible sex-related signals observed in unadjusted analyses were not robust after correction for multiple comparisons and multivariable analysis. This distinction is important because isolated nominal associations may otherwise be overinterpreted as clinically relevant differences. In pediatric emergency care, the present findings support continued reliance on acute clinical manifestations and physiological severity for initial assessment and triage rather than the use of sex as an independent decision-making factor. Larger prospective multicenter studies with greater representation across pediatric age groups and more complete clinical and treatment data are needed to determine whether smaller or age-dependent sex-related patterns exist.

### 4.1. Study Limitations

This study has several limitations. First, its retrospective design entails an inherent risk of information and documentation bias because data were obtained from routine electronic clinical records completed by different healthcare professionals over an eight-year period. Consequently, some symptoms, suspected triggers, latency periods, physiological measurements, and emergency-care variables may have been incompletely or inconsistently documented. Missing data were particularly relevant for suspected-trigger information, which was available for 241 of 368 patients (65.5%), and for TRIPED-GM classification, which was available for 278 patients (75.5%). Analyses were therefore based on available cases, and the possibility that missingness introduced selection bias cannot be excluded.

Second, the study population comprised patients presenting with suspected anaphylaxis rather than exclusively patients who fulfilled all specific Galaxia diagnostic criteria. Although this approach reflects the clinical context in which emergency nursing assessment and triage are performed, it may limit comparability with studies restricted to confirmed anaphylaxis. In addition, the study was conducted at a single tertiary hospital, which may limit the generalizability of the findings to other pediatric populations, emergency departments, healthcare systems, or triage models.

Third, the retrospective records did not consistently contain all physiological and clinical information relevant to the assessment of reaction severity. Individual hemodynamic variables, including blood pressure, heart-rate abnormalities, capillary refill time, and peripheral perfusion, could not be systematically analyzed. Accordingly, PAT shock categories were based on the structured clinical assessment documented at presentation rather than on retrospective reconstruction from individual physiological measurements. Similarly, the records did not consistently provide sufficient detail to determine the persistence or recurrence of vomiting or the intensity of abdominal pain. These limitations should be considered when interpreting cardiovascular and gastrointestinal involvement.

Fourth, the assessment of emergency care was restricted to the measured healthcare-process variables available in the dataset. Detailed information on anaphylaxis treatment, including intramuscular adrenaline administration, time to adrenaline, repeated adrenaline doses, intravenous fluid therapy, bronchodilator use, duration of observation, biphasic reactions, adrenaline autoinjector prescription, and discharge action plans, was not systematically available or was not included in the dataset. The indication, oxygen saturation threshold, delivery device, flow rate, and duration of supplemental oxygen therapy were also not consistently available. Therefore, the present findings should not be interpreted as a comprehensive evaluation of emergency anaphylaxis management.

Fifth, no a priori sample-size calculation was performed because the study included all eligible cases identified during the predefined study period. Several outcomes and subgroups contained relatively small numbers of observations, particularly less frequent clinical manifestations and older pediatric age groups. This reduced statistical power, produced wide confidence intervals for some effect estimates, and limited the ability to detect small or age-dependent sex-related differences. Bonferroni adjustment further reduced the probability of false-positive findings but, given its conservative nature, may also have increased the risk of Type II error. Consequently, non-significant findings should not be interpreted as evidence of equivalence or as proof that clinically relevant sex-related differences are absent.

Finally, boys accounted for a larger proportion of the study cohort, but sex-specific population or pediatric emergency department denominators were not available. Therefore, the present study cannot determine whether the incidence or risk of suspected pediatric anaphylaxis differs between boys and girls. Moreover, because of the observational design, no causal conclusions can be drawn regarding the relationships between sex, clinical presentation, triage acuity, measured emergency-care processes, or suspected triggers.

### 4.2. Implications for Nursing Practice

These findings have relevant implications for pediatric emergency nursing. The absence of robust sex-related differences after adjustment for multiple comparisons reinforces that triage priority and initial management in children with suspected anaphylaxis should be guided primarily by acute clinical presentation and physiological severity rather than by sex, demographic characteristics, or non-acute allergic history. Accordingly, systematic assessment of airway, breathing, circulation, skin, and gastrointestinal manifestations remains essential during triage and subsequent monitoring. Structured pediatric assessment and triage tools can support the early recognition of potentially severe reactions and facilitate timely intervention. Our findings do not support the need for sex-specific modifications to current pediatric triage approaches.

Nurses also play a central role in discharge education and secondary prevention following an anaphylactic episode. Given the predominance of food-related suspected triggers in our cohort, particularly tree nuts, cow’s milk protein, and egg, family education should emphasize avoidance of identified or suspected triggers, early recognition of warning signs, correct use of adrenaline autoinjectors, and the availability of an individualized emergency action plan. These educational interventions should be tailored to the child’s age, developmental stage, previous allergic history, and family context rather than to sex. Overall, the findings support a standardized, symptom-driven approach to pediatric anaphylaxis while highlighting the importance of nursing assessment, continued clinical surveillance, and family education across all pediatric groups.

## 5. Conclusions

In this retrospective cross-sectional study of 368 pediatric patients with suspected anaphylaxis, boys accounted for a larger proportion of cases, and food was the predominant suspected trigger. After adjustment for multiple comparisons, no consistent evidence of overall sex-related differences in clinical presentation, measured emergency-care processes, suspected triggers, or overall triage acuity was found. Although Grade 3 TRIPED-GM assignment was more frequent among boys and remained significant in the category-specific analysis, neither the adjusted global comparison nor the ordinal regression supported a consistent independent association between sex and overall triage acuity. Pediatric emergency assessment should therefore remain primarily guided by acute clinical presentation and physiological severity rather than by sex. Larger prospective multicenter studies are needed to determine whether smaller or age-dependent sex-related differences have clinical relevance.

## Figures and Tables

**Table 1:** Family, personal clinical history and symptoms during the anaphylaxis episode.

*n* = 368	Total *n* (%)	Boys *n* (%)	Girls *n* (%)	OR (95% CI)	*p*-Value	*p*-adj (Bonferroni) *
Personal clinical history						
Rhinoconjunctivitis	53 (14.4)	37 (15.4)	16 (12.5)	0.78 (0.42–1.47)	0.448 *	1.000
Asthma	81 (22.0)	57 (23.8)	24 (18.8)	0.74 (0.43–1.26)	0.270 *	1.000
Food allergy	210 (57.1)	135 (56.3)	75 (58.6)	1.10 (0.71–1.70)	0.665 *	1.000
Atopic dermatitis	97 (26.4)	68 (28.3)	29 (22.7)	0.74 (0.45–1.22)	0.239 *	1.000
Anaphylaxis	69 (18.8)	51 (21.3)	18 (14.1)	0.61 (0.34–1.09)	0.092 *	1.000
Family clinical history						
Rhinoconjunctivitis	44 (12)	32 (13.3)	12 (9.4)	0.67 (0.33–1.36)	0.265 *	1.000
Asthma	28 (7.6)	22 (9.2)	6 (4.7)	0.49 (0.19–1.23)	0.123 *	1.000
Food allergy	26 (7.1)	19 (7.9)	7 (5.5)	0.67 (0.28–1.65)	0.383 *	1.000
Atopic dermatitis	7 (1.9)	5 (2.1)	2 (1.6)	0.75 (0.14–3.90)	0.728 *	1.000
Anaphylaxis	1 (0.3)	0 (0)	1 (0.80)	1.00 (1.00–1.00)	0.348 **	1.000
Skin manifestations						
Urticaria	209 (56.8)	133 (55.4)	76 (59.4)	1.18 (0.76–1.82)	0.465 *	1.000
Cutaneous angioedema	183 (49.7)	118 (49.2)	65 (50.8)	1.07 (0.69–1.64)	0.768 *	1.000
Generalized erythema	114 (31.0)	78 (32.5)	36 (28.1)	0.81 (0.51–1.30)	0.387 *	1.000
Upper airway/oropharyngeal manifestations						
Pharyngeal angioedema	28 (7.6)	19 (7.9)	9 (7.0)	0.88 (0.39–2.01)	0.760 *	1.000
Sialorrhea	4 (1.1)	2 (0.8)	2 (1.6)	1.89 (0.26–13.57)	0.613 **	1.000
Respiratory manifestations						
Dyspnea	200 (54.3)	127 (52.9)	73 (57.0)	1.18 (0.77–1.82)	0.450 *	1.000
Wheezing	86 (23.4)	54 (22.5)	32 (25.0)	1.15 (0.70–1.90)	0.589 *	1.000
Cough	71 (19.3)	50 (20.8)	21 (16.4)	0.75 (0.43–1.31)	0.305 *	1.000
Pharyngeal involvement	56 (15.2)	30 (12.5)	26 (20.3)	1.78 (1.00–3.17)	0.040 *	1.000
Stridor	18 (4.9)	13 (5.4)	5 (3.9)	0.71 (0.25–2.04)	0.522 *	1.000
Chest retractions	16 (4.3)	11 (4.6)	5 (3.9)	0.85 (0.29–2.49)	0.761 *	1.000
Tachypnea	14 (3.8)	7 (2.9)	7 (5.5)	1.93 (0.66–5.62)	0.223 *	1.000
Cyanosis	13 (3.5)	11 (4.6)	2 (1.6)	0.33 (0.07–1.51)	0.135 *	1.000
Dysphonia	10 (2.7)	6 (2.5)	4 (3.1)	1.26 (0.35–4.54)	0.725 *	1.000
Pharyngeal obstruction	6 (1.6)	3 (1.3)	3 (2.3)	1.90 (0.38–9.53)	0.423 **	1.000
Gastrointestinal manifestations						
Vomiting	157 (42.7)	105 (43.8)	52 (40.6)	0.88 (0.57–1.36)	0.564 *	1.000
Abdominal pain	61 (16.6)	39 (16.3)	22 (17.2)	1.07 (0.60–1.90)	0.818 *	1.000
Nausea	20 (5.4)	15 (6.3)	5 (3.9)	0.61 (0.22–1.72)	0.345 *	1.000
Diarrhea	13 (3.5)	12 (5.0)	1 (0.8)	0.15 (0.02–1.16)	0.039 **	1.000
Neurological manifestations						
Irritability	15 (4.1)	11 (4.6)	4 (3.1)	0.67 (0.21–2.15)	0.500 *	1.000
Confusion	5 (1.4)	5 (2.1)	0 (0.0)	1.00 (1.00–1.00)	0.168 **	1.000
Hypotonia	3 (0.8)	3 (1.3)	0 (0.0)	1.00 (1.00–1.00)	0.554 **	1.000
Circulatory manifestations						
Dizziness	7 (1.9)	4 (1.7)	3 (2.3)	1.42 (0.31–6.43)	0.698 **	1.000
Loss of consciousness	5 (1.4)	4 (1.7)	1 (0.8)	0.46 (0.05–4.20)	0.662 **	1.000
Diaphoresis	2 (0.5)	0 (0.0)	2 (1.6)	1.00 (1.00–1.00)	0.120 **	1.000
Livedo	1 (0.3)	1 (0.4)	0 (0.0)	1.00 (1.00–1.00)	1.000 **	1.000

Data are expressed as absolute numbers and percentages. Percentages were calculated using the total number of participants in each group as the denominator (*n* = 368 overall, *n* = 240 boys, and *n* = 128 girls). Clinical manifestations were not mutually exclusive; therefore, percentages within the same organ-system category may exceed 100%. Comparisons between boys and girls were performed using Pearson’s chi-square test (*) or Fisher’s exact test (**), as appropriate. Odds ratios (ORs) with corresponding 95% confidence intervals (95% CIs) are reported. *p*-values were adjusted for multiple comparisons using the Bonferroni correction. In analyses involving zero cell counts, the Haldane-Anscombe correction (+0.5) was applied to enable estimation of ORs and 95% CIs. Statistical significance was set at a two-sided *p* < 0.05. This table consists of the allergic-history and clinical-manifestation comparison family and included 36 comparisons.

**Table 2:** Emergency department assessment and measured emergency-care processes in patients with suspected anaphylaxis, according to sex.

*n* = 368	Total *n* (%)	Boys *n* (%)	Girls *n* (%)	OR (95% CI)	Raw *p*-Value	*p*-adj (Bonferroni) *
Pediatric Assessment Triangle (PAT)						
Stable	256 (69.6)	166 (69.2)	90 (70.3)	1.06 (0.66–1.69)	0.820 *	1.000
Respiratory distress	69 (18.8)	46 (19.2)	23 (18.0)	0.92 (0.53–1.61)	0.779 *	1.000
Respiratory failure	8 (2.2)	5 (2.1)	3 (2.3)	1.13 (0.27–4.80)	1.000 **	1.000
Compensated shock	14 (3.8)	9 (3.8)	5 (3.9)	1.04 (0.34–3.18)	0.941 *	1.000
Decompensated shock	12 (3.3)	8 (3.3)	4 (3.1)	0.94 (0.28–3.17)	0.915 *	1.000
Cardiorespiratory compromise	6 (1.6)	5 (2.1)	1 (0.8)	0.37 (0.04–3.20)	0.669 **	1.000
Central nervous system dysfunction	3 (0.8)	1 (0.4)	2 (1.6)	3.79 (0.34–42.25)	0.278 **	1.000
Measured emergency-care processes						
Shock/resuscitation room	44 (12.0)	29 (12.1)	15 (11.7)	0.97 (0.50–1.88)	0.918 *	1.000
Oxygen therapy	39 (10.6)	29 (12.1)	10 (7.8)	0.62 (0.29–1.31)	0.205 *	1.000
Diagnosis of anaphylaxis	324 (88.0)	214 (89.2)	110 (85.9)	0.74 (0.39–1.41)	0.363 *	1.000
Hospital admission	24 (6.5)	14 (5.8)	10 (7.8)	1.37 (0.59–3.17)	0.464 *	1.000
TRIPED-GM triage classification (*n* = 278)						
Overall TRIPED-GM distribution ^†^	278 (100)	181 (65.1)	97 (34.9)	Cramér’s V = 0.22	0.010 **	0.060
Grade 1	5 (1.8)	2 (1.1)	3 (3.1)	2.86 (0.47–17.39)	0.347 **	1.000
Grade 2	149 (53.6)	89 (49.2)	60 (61.9)	1.68 (1.01–2.77)	0.045 **	0.224
Grade 3	99 (35.6)	77 (42.5)	22 (22.7)	0.40 (0.23–0.69)	<0.001 **	0.005
Grade 4	24 (8.6)	12 (6.6)	12 (12.4)	1.99 (0.86–4.61)	0.104 *	0.521
Grade 5	1 (0.4)	1 (0.6)	0 (0.0)	1.00 (1.00–1.00)	1.000 **	1.000

PAT, Pediatric Assessment Triangle; TRIPED-GM, Five-level pediatric triage system of Hospital General Universitario Gregorio Marañón; OR, odds ratio; CI, confidence interval. Values are presented as *n* (%). Percentages for PAT and measured emergency-care variables were calculated using the full-cohort denominators (*n* = 368 overall, *n* = 240 boys, and *n* = 128 girls). TRIPED-GM percentages were calculated using available-case denominators (*n* = 278 overall, *n* = 181 boys, and *n* = 97 girls; 90 patients with missing TRIPED-GM data, 24.5%). Comparisons between boys and girls were performed using Pearson’s chi-square test (*) or Fisher’s exact test (**), as appropriate. ^†^ The global comparison across the five TRIPED-GM triage levels was performed using a 5 × 2 Pearson chi-square test (χ^2^ = 13.2); Cramér’s V = 0.22. The overall TRIPED-GM distribution constituted one of the six primary emergency department severity and healthcare-process comparisons and did not remain statistically significant after Bonferroni adjustment (raw *p* = 0.010; *p*-adj = 0.060). Category-specific analyses of the five TRIPED-GM grades were exploratory post hoc comparisons and were adjusted separately across the five categories (Bonferroni threshold *p* < 0.010).

**Table 3:** Suspected triggers recorded during pediatric emergency department episodes among patients with available trigger information, according to sex.

Suspected Trigger	Total *n* (%)	Boy *n* (%)	Girl *n* (%)	OR (95% CI)	*p*-Value	*p*-adj (Bonferroni) *
Cow’s milk protein	69 (28.6)	42 (27.1)	27 (31.4)	1.23 (0.69–2.19)	0.479 *	1.000
Egg	52 (21.6)	40 (25.8)	12 (14)	0.47 (0.23–0.95)	0.034 **	0.241
Tree nuts	78 (32.4)	48 (31.0)	30 (34.9)	1.19 (0.68–2.09)	0.534 *	1.000
Fish	15 (6.2)	8 (5.2)	7 (8.1)	1.63 (0.57–4.66)	0.359 *	1.000
Shellfish	8 (3.3)	2 (1.3)	6 (7.0)	5.74 (1.13–29.08)	0.026 **	0.181
Fruit	17 (7.1)	14 (9.0)	3 (3.5)	0.36 (0.10–1.30)	0.107 *	0.751
Medications	10 (4.1)	7 (4.5)	3 (3.5)	0.76 (0.19–3.03)	0.702 *	1.000

OR: odds ratio; CI: confidence interval. Values are presented as *n* (%). Suspected-trigger information was available for 241 of the 368 patients (65.5%), including 155 boys and 86 girls; 127 patients (34.5%) had missing suspected-trigger information. Percentages were calculated using the corresponding available-case denominators (*n* = 241 overall, *n* = 155 boys, and *n* = 86 girls). Patients with missing suspected-trigger information were excluded from these calculations and from the corresponding sex comparisons. Suspected-trigger categories were not mutually exclusive; therefore, individual category counts may sum to more than the number of patients with available trigger information. Comparisons between boys and girls were performed using Pearson’s chi-square test (*) or Fisher’s exact test (**), as appropriate. To account for multiple comparisons, Bonferroni correction was applied across the seven suspected-trigger comparisons, resulting in a corrected significance threshold of *p* < 0.0071 (0.05/7). No suspected trigger remained significantly associated with sex after correction for multiple testing. These seven comparisons constituted the suspected-trigger comparison family.

## Data Availability

Data are available upon contact and authorization from the authors of the study due to privacy or ethical restrictions.

## Supplementary Materials

The following supporting information can be downloaded at https://www.mdpi.com/article/10.3390/nursrep16090310/s1, Table S1: Completeness of the main study variables.

## Public Involvement Statement

No public involvement in any aspect of this research.

## Guidelines and Standards Statement

This manuscript was prepared in accordance with the STROBE guidelines for cross-sectional studies.

## Abbreviations

The following abbreviations are used in this manuscript:

EU	European Union
TN	Tree nut
GDPR	General Data Protection Regulation
IQR	Interquartile range
PAT	Pediatric Assessment Triangle
CMP	Cow’s milk protein
SEAIC	Spanish Society of Allergy and Clinical Immunology
PED	Pediatric Emergency Department
TRIPED-GM	Five-level pediatric triage system of Hospital General Universitario Gregorio Marañón
