# Supplementary material for: Sex-Related Differences in Suspected Pediatric Anaphylaxis in a Tertiary Hospital Between 2015 and 2022: A Retrospective Cross-Sectional Study and Implications for Nursing Practice
Source: Nurs Rep. 2026 Sep 1;16(9):310. doi: 10.3390/nursrep16090310 (PMC13610322; doi:10.3390/nursrep16090310)
Supplement: Supplementary file 1 [file nursrep-16-00310-s001.zip › nursrep-4431990-supplementary.pdf]

Supplementary Table S1. Completeness of the main study variables

Variable	Total sample, n	Available data, n (%)	Missing data, n (%)
Age	368	368 (100%)	0 (0%)
Sex	368	368 (100%)	0 (0%)
Family history of allergic disease	368	368 (100%)	0 (0%)
Personal history of allergic disease	368	368 (100%)	0 (0%)
Clinical manifestations	368	368 (100%)	0 (0%)
Pediatric Assessment Triangle classification	368	278 (75.5)	90 (24.5)
TRIPED-GM triage level	368	278 (75.5)	90 (24.5)
Shock/resuscitation room care	368	368 (100%)	0 (0%)
Oxygen therapy	368	368 (100%)	0 (0%)
Hospital admission	368	368 (100%)	0 (0%)
Final diagnosis	368	368 (100%)	0 (0%)
Suspected allergen	368	304 (82.6)	64 (17.4)
Symptom latency	368	175 (47.6)	193 (52.4)]
